# Impact of Digital Inclusion Initiative to Facilitate Access to Mental Health Services: Service User Interview Study

**DOI:** 10.2196/51315

**Published:** 2024-07-26

**Authors:** Amy Oliver, Ella Chandler, Julia A Gillard

**Affiliations:** 1 Camden and Islington NHS Foundation Trust London United Kingdom; 2 Division of Psychology and Language Sciences University College London London United Kingdom; 3 School of Psychology and Sport Science Anglia Ruskin University Cambridge United Kingdom

**Keywords:** digital exclusion, digital inclusion, video consultation, COVID-19, tablet loan scheme, mental health, telemedicine, digital divide, digital inequality, technology

## Abstract

**Background:**

Digital exclusion, characterized by a lack of access to digital technology, connectivity, or digital skills, disproportionally affects marginalized groups. An important domain impacted by digital exclusion is access to health care. During COVID-19, health care services had to restrict face-to-face contact to limit the spread of the virus. The subsequent shift toward remote delivery of mental health care exacerbated the digital divide, with limited access to remote mental health care delivery. In response, Camden and Islington National Health Service Foundation Trust launched the innovative Digital Inclusion Scheme (DIS).

**Objective:**

This study aimed to examine the impact of facilitating digital inclusion in mental health access. Camden and Islington National Health Service Foundation Trust implemented the trust-wide DIS for service users who were digitally excluded, that is, were without devices or connectivity or reported poor digital skills. The scheme provided access to a loan digital device (a tablet), internet connectivity devices, and mobile data, as well as personalized digital skills support.

**Methods:**

The DIS went live in October 2021 and received 106 referrals by June 2022. Semistructured interviews were conducted with 12 service users to ask about their experience of accessing the DIS. A thematic analysis identified themes and subthemes relating to the extent of their digital exclusion before engaging with the scheme and the impact of accessing a scheme on their ability to engage with digital technology and well-being.

**Results:**

There were 10 major themes. A total of 6 themes were related to factors impacting the engagement with the scheme, including digital exclusion, relationship to the trust, the importance of personalized digital support, partnership working, device usability and accessibility, and personal circumstances. The remaining 4 themes spoke to the impact of accessing the scheme, including improved access to services, impact on well-being, financial implications, and a greater sense of empowerment.

**Conclusions:**

Participants reported an increased reliance on technology driving the need for digital inclusion; however, differences in motivation for engaging with the scheme were noted, as well as potential barriers, including lack of awareness, disability, and age. Overall, the experience of accessing the DIS was reported as positive, with participants feeling supported to access the digital world. The consequences of engaging with the scheme included greater perceived access to and control of physical and mental health care, improved well-being*,* and a greater sense of empowerment. An overview of the lessons learned are provided along with suggestions for other health care settings that are looking to implement similar schemes.

## Introduction

### Background

The emergence of COVID-19 in the United Kingdom led to a rapid transformation in the provision of health services, as it became necessary to reduce face-to-face contact to limit the spread of the virus [1]. As the general population experienced a marked and a prolonged deterioration in mental health, remote care and telehealth practices were rapidly adopted by health care systems to ensure continuity of care [2-4]. Disruption to community and third-sector services further proved detrimental to already at-risk populations, including those with preexisting mental health conditions and severe mental illness or those affected by financial destitution and homelessness [5,6]. As such, the integration of digital solutions into health care became inevitable, with advocates noting greater flexibility to meet individual needs and digital transformation as key to implementing the National Health Service (NHS) long-term transformation strategy [7-9].

However, concerns were raised by practitioners and service users as to the feasibility of engaging in telehealth interventions or other forms of digital health care delivery. These included the ability of staff and service users to navigate devices and platforms, the lack of physical or financial resources to obtain technology, poor connectivity, concerns around cybersecurity, and physical privacy when providing or receiving telehealth care in a home environment [1,8,10,11]. Digital exclusion, characterized by lack of access, connectivity, or digital skills, is another domain that presents a challenge to the remote delivery of health care with marginalized groups particularly affected [12]. This includes older adults [3], homeless people [13], immigrants [14], people with disabilities [12], and individuals with severe mental illness (eg, psychosis) [15].

The term *digital divide* conceptualizes this inequality, differentiating between those who have access and those who do not [16]. It consists of 3 levels [17]: *lack of access* to information and communications technology (ICT), for example, computer, tablet or mobile phone devices, or data; *lack of digital skills or confidence*; and *lack of awareness, or interest*, in the opportunities digital technology can provide [18]. The 3 strands of the digital divide are not linear, but rather they are interactional and reciprocal; one may have a high level of digital ability; however, without an ICT device, one cannot use these skills, while the likelihood of being digitally excluded is exacerbated by societal inequities, and the lack of access can itself perpetuate marginalization [19-21]. The relationship between digital exclusion and preexisting inequalities thus limits individuals’ ability to participate in the digital world; can lead to greater social isolation [20]; and prohibit access to education, training, and employment opportunities [22] or, critically, access to mental and physical health services [13].

With the shift toward remote delivery of mental health services exacerbating the digital divide in the wake of COVID-19, the Camden and Islington NHS Foundation Trust (C&I NHS FT) launched the innovative Digital Inclusion Scheme (DIS) to support digitally excluded service users accessing community mental health services. The scheme provided access to a loan ICT device (a tablet), internet connectivity devices, and mobile data, as well as personalized digital skills support to enhance literacy and confidence. This scheme aimed to build on lessons learned from previous initiatives, which had predominantly focused on increasing digital skills and competency to bridge the digital divide [3,11,21,23,24]. However, the lack of physical access to resources remained an immediate concern for those affected by digital exclusion [25]. Similarly, interventions restricted in time (eg, NAViGO) [12] or limited in reach, for example, only available for a small number of inpatients [4]. Interventions also tended to support organizations rather than individuals (eg, Digital Communities Wales) [26] or link together various community organizations rather than providing holistic support, for example, 100% Digital Leeds [27]. Pertinent was also the marked absence of robust evaluation regarding the impact of such schemes.

### Objective

Following its launch in October 2021, this evaluation therefore aimed to understand the impact of the DIS on service users, as well as to identify the barriers and facilitators to implementing such schemes in future. Specifically, it aimed to (1) summarize the project and communicate the lessons learned to date, (2) understand the barriers and facilitators to accessing digital technology within a DIS within a population of mental health service users, and (3) explore the impact on service users who used the scheme and any impact it had on their well-being.

It is anticipated this evaluation will inform clinicians, service planners, and commissioners as to whether this type of scheme is feasible and acceptable in the context of an NHS trust and provide an example design to those both within the NHS and wider community groups.

## Methods

### Design

This evaluation used an intervention mixed methods framework, combining quantitative and demographic data from the referral form and baseline questionnaire (part 1) and semistructured qualitative interviews to understand the impact of the scheme (part 2). This evaluation encompasses referrals received between October 2021 and June 2022.

### Setting

C&I NHS FT is a large inner-city NHS trust, which provides mental health and substance misuse services. Due to the COVID-19 pandemic and social distancing requirements, most NHS services transitioned to delivering treatment remotely, leaving many service users without continued access to care. In response, C&I NHS FT established a DIS to support service users at risk of digital exclusion. The scheme went live in October 2021 and received 106 referrals by June 2022.

### Initiative

The DIS provided a response to the 3 key areas of digital exclusion: provision of devices; connectivity; and support to build skills, confidence, and motivation to engage with digital technology. To this end, the trust purchased 185 Samsung Galaxy Tablets, of which 25 supported 4G connectivity in addition to being Wi-Fi enabled. Additionally, 100 SIM cards, each with 20 GBs of free data for 6 months, were obtained from Vodafone as part of the “charities.connected” initiative [28].

The project also extended preexisting partnerships with the UK-based not-for-profit Jangala [29] and AbilityNet, a UK charity [30]. Jangala loaned C&I NHS FT 30 Get Boxes, which is a mobile router that operated using a SIM card and provided simultaneous connectivity for up to 10 devices. AbilityNet used their web-based resources and network of community-based volunteers to provide 1:1 tailored digital support. The trust also invested in 185 protective cases and 20 Bluetooth keyboards to support accessibility needs.

A digital inclusion officer (DIO) was employed by the trust on a 0.4 full time equivalent basis to facilitate the scheme. The DIO was embedded within a clinical team and line managed by a clinical psychologist who could provide risk and safeguarding advice where needed. A digital workspace was developed on ServiceNow (ServiceNow, Inc) [31], which was integrated into the wider IT infrastructure. This was used by the DIO to administer the scheme, operating as both a stock inventory and log of all loans, as well as providing a digital workflow to track the loan process.

### Pathway of Support

**Figure 1:**
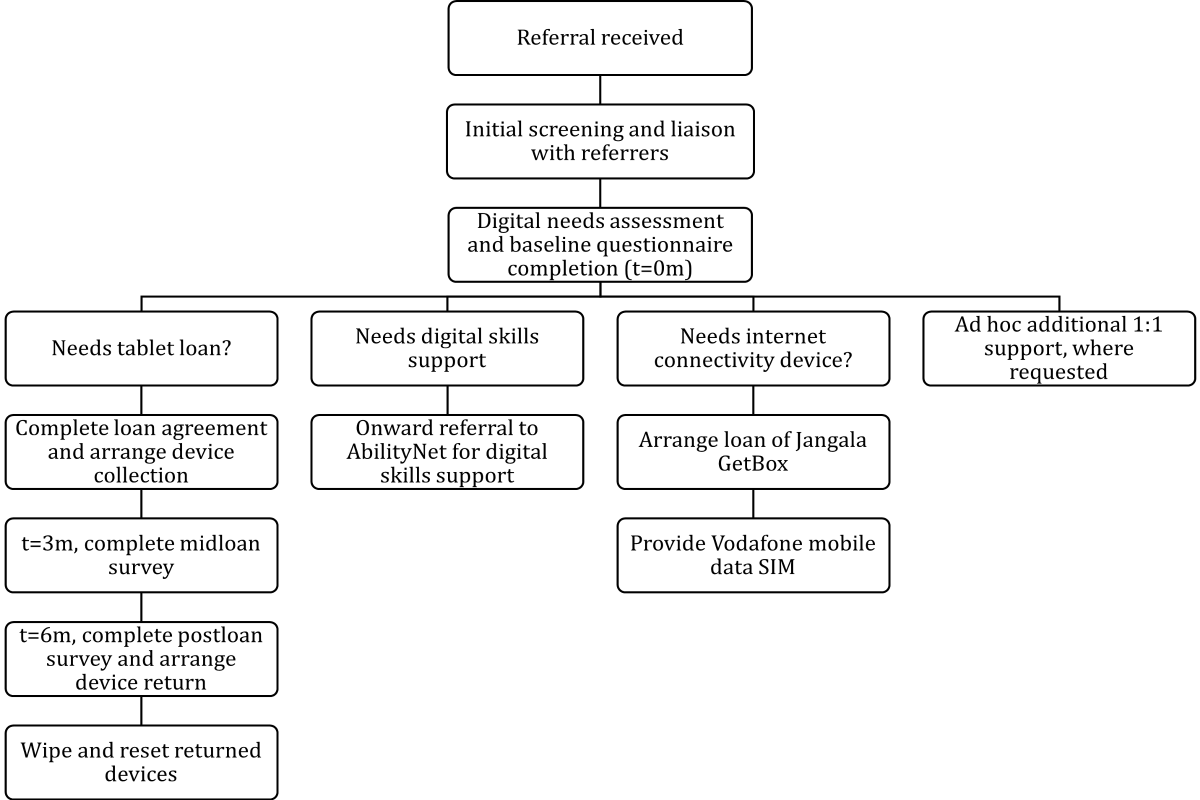
Digital inclusion referral pathway.

Service users were referred via a web-based form by the C&I staff members and screened by the DIO for eligibility and suitability (see Figure 1 for referral pathway). To be eligible for the scheme, service users had to be (1) currently accessing mental health services within C&I NHS Foundation Trust and (2) referred by a mental health professional or key worker who worked within the trust. In addition, they had to be at risk of digital exclusion, experiencing (1) lack of access to digital devices, (2) lack of internet connectivity, and (3) lack of skills or confidence in accessing digital technology.

Following the receipt of the referral, the DIO would screen the referral for any risk information and liaise with the referrer before contacting the service user to complete an initial telephone or in-person digital needs assessment. The digital needs assessment involved completing a baseline questionnaire (Multimedia Appendix 1) to understand individuals’ digital skills and confidence in accessing digital technology. The DIO would also clarify service users’ current digital needs and confirm eligibility for the scheme. Once service users opted into engaging with the scheme, including the receipt of a tablet loan, the DIO would discuss the practicalities of the loan, as well as the terms of the loan agreement, which detailed that service users were able and encouraged to use the devices for personal use under the conditions of the loan agreement.

The DIO then provided the service user with a tablet loan, a connectivity device, or free mobile data SIMs and facilitated an onward referral to the partner organization AbilityNet for digital skills support. The DIO would also provide further ad hoc support around digital skills training or other digital resources. The DIO would meet service users within a familiar environment (ie, home visits) or within the trust premises, considering risk and safeguarding concerns, where identified.

Over the course of the loan, service users were contacted by telephone or email on 2 occasions to complete a midloan survey after 3 months and a postloan survey after 6 months, unless referrers or service users requested a loan extension. At the end of the loan period, the DIO would liaise with the service user and referrer to arrange the return of the device and assess further needs. Throughout their engagement with the scheme, service users were also encouraged to bring any concerns or questions about their devices to the DIO for ad hoc support. The DIO could also facilitate a referral for additional digital skills training through the partnership with AbilityNet at any point throughout the loan period. Once the device had been returned, the DIO would liaise with trust IT services to wipe and reset the device for a new loan cycle.

### Participants

#### Part 1

In total, 106 service users were referred to the scheme between October 2021 and June 2022. Service users were considered to have engaged with the scheme if they had completed a baseline questionnaire (Multimedia Appendix 1) following the referral. Of the 106 service users referred, 83 (78.3%) completed the baseline questionnaire, and the remainder 23 (21.7%) either did not wish to take part in the scheme or did not engage, for example, not responding to phone calls or SMS text messages. The inclusion criteria were being a current user of mental health services within C&I NHS Foundation Trust, over the age of 18 years, and at risk of digital exclusion.

#### Part 2

**Figure 2:**
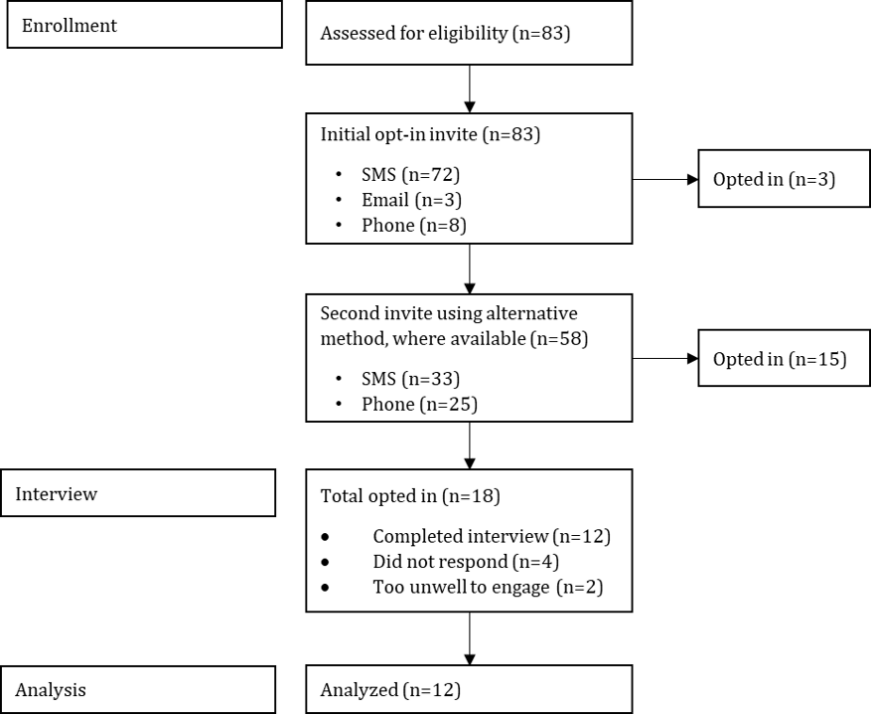
Recruitment pathway.

The study used convenience sampling of individuals who had prior engagement with the DIS by completing, at minimum, the baseline questionnaire during the initial digital needs assessment. As such, 83 service users were eligible to take part in the formative service evaluation and were invited. A total of 12 participants were recruited via SMS text message, email, or telephone and were subsequently interviewed (Figure 2). Recruitment ceased once data saturation had been reached. Efforts were made to invite service users who chose not to engage with the scheme, and all the participants were informed that their contribution to the service evaluation would have no bearing on their current or future engagement with the scheme, which aimed to reduce the risk of bias.

### Measures

#### Part 1: Baseline Questionnaire

The baseline questionnaire was derived from *The Digital Inclusion Evaluation Toolkit: Bank of Outcomes, Indicators and Survey Questions* [32] and consisted of 10 items, including questions such as “Do you consider your digital skills to be good, average, or poor?” “How confident are you in using digital technology?” and “Is there anything that limits you from using the internet at the present moment?” (Multimedia Appendix 1).

The questionnaire aimed to ascertain service users’ self-reported level of digital skills and confidence in accessing and using digital technology, as well as their frequency and type of use. It also provided insight into what factors led to service users being digitally excluded by reporting what limited their use of the internet and whether COVID-19 had made accessing the internet and using digital technology more important. It also established what resources they required from the scheme to meet their digital needs. service users were able to indicate whether they needed assistance with acquiring equipment, accessing data connectivity, and support with using digital technology and digital resources on the internet.

#### Part 2: Interview Schedule

A semistructured interview schedule (Multimedia Appendix 2) was created to obtain qualitative data to understand the impact of the DIS. Each interview lasted between 30 and 40 minutes and was conducted by the same researcher (AO) to minimize interrater variability [33]. The questions were based on the initial aims and perceived outcomes of the DIS and preexisting literature [34] while also following guidance on good practice for service evaluations [35] and ensuring any potential ethical issues were considered [36]. Interview topics covered factors contributing to digital exclusion, questions relating to primary outcomes, including eliciting the perceived barriers and facilitators to engagement with the scheme (eg, “What helped or hindered you in engaging with the support offered by the scheme?” and “What changes would you make to improve the scheme?”), as well as questions relating to their experience of engaging with partner organizations, such as AbilityNet or Jangala. The interviews also attempted to elicit potential secondary outcomes, including but not limited to the impact of engagement on well-being, social interactions, ability to access C&I NHS FT or other health services, leisure, finances, education, and employment opportunities. Where applicable, participants were also asked about their experience of accessing the partnership support available, such as that offered by AbilityNet.

### Procedure

#### Part 1

Baseline demographic, digital needs, and digital literacy information was obtained through the referral form and baseline questionnaire, which was carried out by the DIO with the service users’ consent, upon referral to the scheme.

The Initiative section provides further details on the baseline digital needs assessment.

#### Part 2

A link to an evaluation opt in form was sent to all eligible service users if a mobile number had been provided in the initial referral. An email was sent to those who did not provide a telephone contact and for those with only a landline number, and the invitation to the those who chose to participate were contacted by their preferred method, whereby the project was explained, and interviews were scheduled.

Before the interview, participants were provided an information sheet (Multimedia Appendix 3). Further demographic information was obtained after the main interview, and participants were given opportunities to ask any questions. The information sheet also included guidance on further support available should any questions or concerns arise after the session.

The interviews were recorded using the Microsoft Word voice recorder. Interviews were automatically transcribed verbatim by Microsoft Word and anonymized by removing personally identifiable content. The final transcription was checked against the original audio recording for errors and corrected manually by the first author (AO).

### Statistical Data Analysis

#### Part 1

The baseline questionnaire contained self-reported data of participants’ digital skills, confidence, and literacy, as well as current engagement with digital technology. Quantitative data from the referral form and baseline questionnaire was analyzed using SPSS Statistics (version 28.0.0.0; IBM Corp) and JASP (version 0.16.3; JASP Team, 2022) using descriptive statistics.

#### Part 2

Thematic analysis [37] was chosen to explore the qualitative experience of service users accessing the DIS from a realist approach [38]. Each interview was transcribed by the main researcher and checked by another (JAG). Each transcript was reviewed before coding the data using a combination of inductive and deductive approaches [37], deriving codes from the data within the primary areas of interest. Transcripts were repeatedly checked against the emergent themes before a list of themes and subthemes were collated through discussion between 2 authors (AO and JAG). The analysis was conducted using NVivo 12.7.0 software (QSR International) before the results were synthesized into a thematic analysis.

### Ethical Considerations

Ethical approval was obtained from the Anglia Ruskin University School Research Ethics Panel (ETH2122-0635; July 29, 2022) and the C&I NHS FT Clinical and Information Governance Department (July 12, 2022). Informed consent was obtained from the participants before the interview (Multimedia Appendix 4)

## Results

### Part 1: Baseline Digital Needs and Demographics

Of the 83 service users who completed the baseline questionnaire, 52% (n=43) were female, and 48% (n=40) were male, with a mean age of 52.42 (SD 13.47) years.

Almost all reported requiring a device (n=78, 94%), with approximately half reporting a need for internet connectivity (n=45, 54%) or digital skills support (n=57, 69%). One-third of referrals (n=31, 37%) indicated requiring support across all domains of need.

Of those reporting a lack of devices, 77 (93%) were provided with a loan tablet; 40 (48%) were provided with a Vodafone SIM card to access mobile data; and a further 15 (18%) were given SIM-enabled Jangala Get Boxes, which acted as a stand-alone internet connectivity device. A total of 29 (35%) service users requiring extra digital skills support were referred to AbilityNet by the DIO. While the scheme could provide additional 1:1 support with the DIO, some service users were also signposted to a Recovery College [39] course focused on improving digital skills, cofacilitated by the DIO or other digital support delivered by services across the C&I NHS FT or in the wider community.

At baseline, service users reported various factors that limited their ability to access the internet (Table 1), with the lack of devices and lack of knowledge and skills in using devices representing the most common barriers to accessing the internet. Those who did access the internet did so for a variety of purposes (Table 1), with more than half of respondents (n=43, 52%) indicating entertainment as a primary driver. Overall, 70% (n=57) of service users reported that access to digital technology and the internet had become more important as a result of the COVID-19 pandemic (Table 1).

**Table 1:** Participant characteristics and baseline digital skills, confidence, and use before participation in the Digital Inclusion Scheme (n=83).

Characteristic		Values
**Gender, n (%)**		
	Female	43 (52)
	Male	40 (48)
**Age^a^ (y)**		
	Overall, mean (SD)	52.42 (13.47)
	Range	21-88
	Female, mean (SD)	53.83 (14.36)
	Male, mean (SD)	50.86 (12.41)
**Ethnicity, n (%)**		
	Asian or Asian British	2 (2)
	Black or Black British	12 (14)
	Kurdish	1 (1)
	White	50 (60)
	Mixed	4 (5)
	Not known	14 (17)
**Level of digital skills, n (%)**		
	Good	13 (16)
	Average	26 (31)
	Poor	44 (53)
**Confidence in use of digital technology, n (%)**		
	Extremely confident	7 (8)
	Somewhat confident	20 (24)
	Neutral	13 (16)
	Somewhat not confident	19 (23)
	Extremely not confident	24 (29)
**Frequency of internet use, n (%)**		
	Every day	36 (43)
	A few times a week	13 (16)
	A few times a month	6 (7)
	Never	28 (34)
**Self-reported limits on internet use, n (%)**		
	I did not have a device	40 (48)
	I struggle to know how to use my device	39 (47)
	Other	34 (41)
	I did not have an internet connection	28 (34)
	My device was not good quality	14 (17)
	I had an internet connection but it was too slow	6 (7)
	There was no reason for me to use my device	4 (5)
	I do not have time	4 (5)
	None of my friends and family are online	0 (0)
**Self-reported use of digital technology, n (%)**		
	For entertainment (ie, to watch videos)	43 (52)
	Other	36 (43)
	To speak to friends and family	24 (29)
	To stay up to date (ie, read the news or Twitter)	22 (27)
	To access other physical and/or mental health services	23 (28)
	To do online shopping	20 (24)
	Nothing, I didn’t use it	20 (24)
	To speak to my GP	20 (24)
	To learn	18 (22)
	To speak to my clinician, care coordinator, or service	11 (13)
	To access other online mental wellbeing support	7 (8)
	To earn money	3 (4)
	To access social services	3 (4)

^a^Age unknown: n=5 (male: n=3; female: n=2).

### Part 2: Semistructured Interview Results

#### Overview

A total of 12 semistructured interviews were conducted between July 6, 2022, and July 14, 2022, either over the phone (n=11) or via Microsoft Teams (n=1). This obtained the qualitative data focusing on the service users’ experience of the DIS as well as basic demographic information (Table 2).

The results from the semistructured interviews are presented in subsequent sections. There were 10 major themes in total. These 10 themes could be clustered into two superordinate themes: (1) factors impacting participant’s engagement and (2) the impact of accessing the scheme. For the former (1), the 6 major themes that were shared among participants included digital exclusion, relationship to trust, the importance of personalized digital support, partnership working, device usability and accessibility, and personal circumstances. In the latter superordinate theme (2), we identified 4 major themes: improved access to services, impact on well-being, financial implications, and a greater sense of empowerment.

**Table 2:** Demographics of participants (n=12).

Characteristic			Values
**Age (y), n (%)**			
	25-34	2 (17)	
	35-44	2 (17)	
	45-54	1 (8)	
	55-64	4 (33)	
	65-74	1 (8)	
	75-84	2 (17)	
**Gender, n (%)**			
	Male	5 (42)	
	Female	6 (50)	
	Gender fluid or gender queer	1 (8)	
**Ethnicity, n (%)**			
	Asian or Asian British (Bangladeshi)	1 (8)	
	Black or Black British (African)	1 (8)	
	Black or Black British (Caribbean)	1 (8)	
	White British	5 (42)	
	White Irish	2 (17)	
	Prefer not to say	1 (8)	
	Other (White British Traveller)	1 (8)	
**Employment status, n (%)**			
	Unemployed	4 (33)	
	Unable to work	4 (33)	
	Student	1 (8)	
	Retired	2 (17)	
	Unknown	1 (8)	
**Highest level of education, n (%)**			
	Secondary school	4 (33)	
	Sixth form college or further education (A-levels, BTEC^a^, etc)	4 (33)	
	Higher education or university (diploma, bachelor’s degree, etc)	2 (17)	
	Postgraduate education	2 (17)	
**Disability or long-term physical or mental health condition, n (%)**			
	Yes	11 (92)	
	Prefer not to say	1 (8)	
**Relationship status, n (%)**			
	Married or civil partnership	2 (17)	
	Living with someone, but not married or in civil partnership	1 (8)	
	Single	8 (67)	
	Widowed	1 (8)	
**Household income over last year (£^b^), n (%)**			
	<10,000	7 (58)	
	10,001-20,000	3 (25)	
	Prefer not to say	2 (17)	
**Support accessed, n (%)**			
	Tablet loan (C&I NHS FT^c^)	12 (100)	
	Internet connectivity router (Jangala)	4 (33)	
	Mobile data (Vodafone)	7 (58)	
	Digital skills support referral (AbilityNet)	2 (17)	
	Other digital support or skills training (including DIO^d^)	6 (50)	
	Keyboard	3 (25)	

^a^BTEC: Business and Technology Education Council.

^b^Average exchange rate in July 2022: £1 GBP=US $1.2.

^c^C&I NHS FT: Camden and Islington National Health Service Foundation Trust.

^c^DIO: digital inclusion officer.

#### Engagement With Scheme

#### Digital Exclusion

Of the 12 participants, 3 (25%) participants reflected on how COVID-19 had made digital technology more important, with 7 (58%) participants reporting that they felt “forced” to learn how to use technology. They noted that before accessing the scheme, the increasing reliance on digital technology had resulted in them feeling isolated and excluded:

It’s really important, especially since COVID.... Everything has moved online.

P1

I got people bothering me...she said you gotta learn how to do emails and stuff like that.

P2

It’s just something we, we never could do before. [We felt] isolated, left behind.

P10

I remember using the clunky old computers with the floppy disks and all of that. Very dim memories. I didn’t use [new technology] particularly well...I kind...of felt excluded in the sense that [these were] things I couldn’t possibly buy over the years. I didn’t have the capacity to keep up to speed with it because I let it slip for so many years.

P11

A total of 5 (42%) participants reported that the sudden digital transformation in the wake of COVID-19 left them unable to access crucial mental or physical health support, information (eg, pertaining to health care or local services), and government and banking services, as well as being unable to pay bills:

I have to transfer onto the Universal Credit scheme. That was another reason for really having to get to grips with the IT because you can only access that Universal Credit thing via IT, you can’t do it postally anymore. So that was kind of a worry in the back of my mind, I knew it was coming up at some point, and some point this year I’ll have to fill out one of these multi page online forms which I’m not looking forward to.

P11

Anything to do with art I am kind of interested in, and yoga. And I said, well I can’t...because my camera doesn’t work...an external camera or...external mic, none of that worked.

P6

Moreover, participants reported that their digital exclusion before accessing the scheme was driven by a lack of suitable devices or internet access, as well as defective or unsuitable equipment. One-third (4/12, 33%) of the participants reported that their device did not work or was not fit for purpose when needing to engage in education, employment, or therapy. It was noted that, as a result, they were unable to engage with web-based mental health appointments offered, with some participants opting for telephone consultations instead:

Before I saw my therapist, I was being assessed, but because of COVID, I couldn’t go out. My computer didn’t work. So...in the end it was all done on the phone with another therapist.

P6

A major impact I was finding it difficult to study. I’m a student, so it makes a big impact. I had a mobile phone, but I wasn’t able to use anything like for typing and submitting assessments and assignments and things like that.

P1

In addition, the level of self-reported competency was low before accessing the scheme. One-third (4/12, 33%) of participants reported never having used digital technology, whereas 7 (58%) struggled despite having some experience with technology though limited to laptops or computers. Only 1 (8%) participant reported being proficient, but that they were unable to afford their own device. Digital confidence was also low, with 10 (83%) participants reflecting on the challenges they faced using devices, even those who professed to have had some experience:

I would say familiar. But I wouldn’t say expert, you know what I mean? I still get lost sometimes.

P6

I never used anything like that [loan tablet] before in my life. I used to have a landline. So that’s the most...we used as communication.

P12

#### Relationship to the Trust

The relationship with trust staff was key in facilitating or encouraging the service users’ engagement with the scheme. All 12 participants were unaware of the existence of the scheme before being referred by staff members; however, there were differences in the motivation to engage with the scheme. One participant found that accessing the scheme helped to foster their own agency and independence:

I knew [about the DIS], from my key worker, ‘cause I didn’t know about you guys. [I engaged because] I couldn’t keep on going to my key worker with every problem I had.

P4

A total of 5 (42%) participants acknowledged they felt more inclined to engage with the scheme due to it being recommended by their health professional:

It was through my care worker.... I remember her phoning me up and saying I’ve got you a tablet...so I said, well all that sounds great. I’ll, I’ll proceed with, with this thing, you know, and it’s turned out pretty decent for me to have one, you know.

P9

I think without being pushed into it [by the referrer], we might never have actually...ever thought we would possibly even attempt to use [a device], you know.

P7

However, for one participant, being referred by their health professional led to them feeling obliged to accept the help, despite the benefits:

I mean, all these things have a kind of psychological element, so when you’re...offered something by your...long-term nurse or therapist, you’re more psychological inclined to please them and say yes, I’ll try that, it’s a good idea.

P11

Nonetheless, 2 (17%) participants reported feeling more positive toward the scheme, as it was a service provided by the NHS, rather than a “corporation” (P9), with another participant reporting that they “would feel more vulnerable if Barclays had given it [the device] to me.” Similarly, another participant felt that accessing NHS support and resources through the technology by the scheme was safer in general:

I also like the fact that it [is an NHS scheme], ‘cause I doubt very much that Joe Public can get access to what I’m getting access to through [the NHS]. So, it’s helping [the NHS] to help me, you know?

P6

#### Personalized Digital Support

All participants were positive about the role of the DIO, explaining that they felt “safe” (P2) and that the process was “easy” and the DIO “supportive” (P3) and, overall, worked “extremely well” (P7). Participants appreciated that the DIO was always “available to be contacted” (P11) if there were any problems:

I liked [the DIO]. [They were] very friendly and approachable, and made it very easy because, as I said I’ve got agoraphobia and mental health and stuff, so when I’m accessing services or things, I find it quite difficult, although I’m quite chatty on the, outwardly but, I do like self-judge and stuff quite a lot so, yeah, [they are] just, like, [they are] friendly, [they] made me feel at ease, so it wasn’t a traumatic experience...

P8

Similarly, P8 felt that the support offered was relevant and tailored to their needs:

I think it was quite thorough really, because [they] offered an extension of help and stuff if I needed it...and there were extra apps and stuff on the tablet that were, I felt like they were tailored towards what I had said, like the PTSD [posttraumatic stress disorder] thing, so and I think you mentioned that, that possibly your IT department would put apps on there so...so actually, I think you’ve done that quite well actually.

P8

However, 7 (58%) participants reported needing further digital support to use the device, with 1 participant finding the amount of web-based content “daunting” (P11). In total, 5 (42%) participants identified needing additional support from the DIO, with 6 (50%) receiving digital skills support from external partners, such as AbilityNet or community groups. Despite this, 10 (83%) reported still feeling unsure or uncertain; for example, P7 worried that they “might press the wrong buttons” and resorted to trial and error instead of asking for help. One participant reported feeling uncomfortable asking for further support:

I guess I didn’t want to be too pushy about it. I didn’t know what the boundaries were.

P11

P11 also described feeling that the scheme assumed a higher level of digital competency, which they perceived as impacting on the level of support provided:

That’s a criticism that should be levelled at lots of IT schemes, sometimes they assume too much at the beginning. It was even true of the IT skills course at [the C&I NHS FT service], just a lot was assumed at the earliest stage.

P11

The service received by the participants was also at times delayed by the staffing arrangements of the scheme, with one participant’s ability to join a web-based course affected by the sole DIO being on leave:

I’m still waiting for the next art class to start because I did one session, and they were all very happy and then I got an email saying they’re not happy. The other participants. And I did start quite late, but I mean that wasn’t quite my fault as I couldn’t get hold of the...tablet for ages.

P6

#### Partnership Working

A further essential component of the scheme organization was the partnerships established with external organizations, including AbilityNet for personalized digital support, Jangala for connectivity devices, and Vodafone for SIM cards with free data.

While 1 (8%) participant described the partnership support as “invaluable” and the connectivity device provided by Jangala as “simple” to use, participants were nonetheless more inclined to seek additional support from the DIO (5/12, 42%), as opposed to the designated partner organization (2/12, 17%). For example, while P7 reported that the telephone support provided by the AbilityNet volunteer was “helpful,” they also noted limitations:

I don’t know what I could say about [external support] really, because...I just think that it was very short and quite brief...it’s really not enough time to get things to sink in..., we just need that bit more help like that...just if it could have continued for even for four or five weeks, once a week or once a fortnight, or just something like, that would have been would have been very helpful.

P7

It was just...so basic, I couldn’t say if it was brilliant or not brilliant. It worked. What they said was correct...[But] well you know me with this phone, it’s often turned off...So, it just never matched up.

P11

#### Device Usability and Accessibility

The tablets were described as “easy to set up” (P3) and “helpful” (P1). The physical flexibility of a tablet was also cited as a positive (eg, as it allowed the tablet to be used outdoors or facilitate engagement with therapeutic services from the safety of their home environment). For example, P3 explained how accessing a portable device, such as the loan device, enabled them to participate privately in group therapy:

I didn’t feel as comfortable, I would say, to participate in group therapy without it, without having that privacy element [physically being able to move the tablet to a private space] in it. And so, when I did join via video link it did...I felt more inclusive, and you know able to participate properly in the group.

P3

The additional items available to loan (eg, a keyboard and case) were also reported to be “a massive help” (P8). However, 2 (17%) participants raised concerns about the restrictions placed on the loan device by C&I NHS FT for security purposes. As one participant explained:

I just didn’t like the fact that I couldn’t access some of the services... due to it being sort of owned by the trust. With Google and Google services and things like that.

P1

Other negatives of using a tablet included it being “quite heavy in weight” (P9) and not as practical, or familiar, as a computer:

I think it can be difficult to edit PDF’s, especially on tablets, it’s not quite as straightforward as a computer.

P4

Concerns around the safety or implications of use were also cited as barriers to fully using the loan device. In total, 2 participants did not engage with the loan device once it had been loaned to them, with one instead opting to use an older device provided by their daughter in case they would damage the equipment in any way:

We were quite nervous about getting [a tablet] because we were worried that we might press the wrong buttons, we might do something wrong.

P7

I was afraid to, you know, in case I’d mess it all up. So, my daughter said just use mine and if you if you mess it up, you know what I mean, ‘cause it’s old.

P5

One participant also reported misunderstanding the terms of the tablet loan, limiting their use, believing that the tablet was “not something you use for personal use” (P3).

A total of 5 (42%) participants expressed being worried that in accessing digital technology, they may become more vulnerable to fraud, cyberattacks, or hackers accessing their personal or private information. Consequently, some participants limited their use of the loan device due to concerns around web-based security and privacy, for example:

I’d be very nervous of putting in a debit card number and buying something online, I’m thinking somebody is going to scam me here, something’s going to go wrong. I’m still nervous of that.

P11

I just don’t want anything personal or private on that because it’s not actually mine.

P6

#### Personal Circumstances

In general, disability and poor mental health affected the participants’ ability to engage with the scheme and the digital world more generally. A total of 10 (83%) participants reported having a disability, and nearly half (5/12, 42%) reported mental health difficulties that affected their daily lives and impacted their ability to engage with the scheme:

It’s me, personally, not the service available.... I just lost confidence in everything.

P5

In total, 2 (17%) participants reported difficulties with reading and writing, which hindered their ability to access the scheme or loan device, despite acknowledging its potential benefits:

It would help an awful lot of people; you know what I mean. But not in the situation I am in.... It’s my spelling ability [that hindered me in engaging with the tablet]...You’ve got to kinda spell things.

P12

Beliefs that older age negatively affect technology use were noted by a third of participants (4/12, 33%), both as a causal and maintaining factor for digital exclusion. In total, 2 participants stated a preference for not engaging with technology due to their age:

I grew up without, before that technology came out. So, I’m in my 50s now and I’d prefer to stay in my old school world.

P9

At my age, what do I want a computer for?

P12

A total of 3 (25%) participants expressed feeling motivated to access the digital world despite this. However, the need for additional support to overcome the perceived age-related difficulties in learning to use technology was also highlighted:

You know as you get older, obviously it’s a bit more difficult to pick things up, and you know you need a little bit more than a couple of, sort of, half an hour or even an hour session. I know you can learn by just using it, but you really do need a bit more help in actually using it, you know.

P7

I do find tablets a little bit difficult because I think it might be my age.... I find I’m a little bit disconnected with like iPad and things like that. I find it easier to use computers.

P8

#### Impact of Accessing Scheme

#### Access to Services

The scheme provided “access to the digital world” (P8), with 4 (33%) participants reporting that they had accessed C&I NHS FT services as a direct result of the scheme. For example, P6, had previously struggled to manage their posttraumatic stress disorder symptoms during lockdown and described the benefits of being able to attend web-based yoga classes using their loaned tablet, rather than attending in person:

Communicating with people is becoming increasingly difficult for me. But I feel very comfortable at home, and this has nothing to do with COVID. This is just me shutting down, basically. So being able to stay within my own home environment, safe environment, nobody knows where I live. But I have access to yoga...because of who I am...the very idea of going to a place, in a room with a lot of strangers and try and do yoga or tai chi in a mask is just “no, I’m sorry.”

P6

Improved access to education and employment was noted, with 5 (42%) participants attending web-based courses or using their device to facilitate their studies following engagement with the scheme:

It has given me a sense of, you know, of other possibilities and things like that. It’s kind of, you know, got me out of the house and all that [to attend a course].

P11

A total of 10 (83%) participants also acknowledged the necessity of digital technology and the access it provided. For example, P1 reported the tablet to be an “essential item,” and P7 found they frequently relied upon it:

The first thing I, I would look for if I wanted help, or wanted to look for an organisation, I would look on [the tablet] straight away, you know, and ask them what I wanted and up comes an awful lot of options, you know.

P7

#### Impact on Well-Being

A total of 10 (83%) participants reported that the scheme had a positive effect on their well-being. Accessing the scheme had improved participants’ mood, built confidence and self-esteem, relieved stress, aided sleep, provided “comfort” (P9), and proved to be a distraction from anxiety. A total of 9 (75%) participants also reported feeling included, more connected, and less isolated; no longer the “odd ones out” (P7) and being able to “re-access parts of my old life via things like social media” (P8):

In my general well-being, it’s actually good for me. I’ll give you 100%, I’ll tell you that much. Like literally I go home now when I finish my course at 4, I just go down there and then put music on and start cleaning the house.

P2

It also provided opportunities for entertainment (eg, browsing the internet, playing chess, or listening to music); staying up-to-date (eg, watching the news); and social interactions (eg, video calling friends or using Facebook to keep in touch with others):

Well, maybe I listen to music. Maybe I listen to something educational, you know depends on what mood I’m in, you know.

P4

One participant felt that accessing the tablet had *“*open[ed] up the world to me” (P6), as they did not feel comfortable interacting with people outside of their home:

Communicating with people is becoming increasingly difficult for me. But I feel very comfortable at home, and this has nothing to do with COVID. This is just me shutting down, basically. So being able to stay within my own home environment, safe environment, nobody knows where I live.

P6

However, 3 (25%) participants also expressed potential negative impacts on well-being. For example, P11 expressed concern around using the tablet “too much” and described how having access to a tablet and the digital world also led to feelings of disconnection and isolation, noting that it can be “antisocial...sitting alone with a screen” where “you become wrapped up in [an]...internal and isolated world”:

I get a call from someone to come on up a coffee or something and say no I’m do[ing] this online...because I wasn’t, you know, really au fait with all the IT skills, I thought no no no, I don’t wanna lose my thread and lose what I’m on here...So, there is an addictive aspect. It’s really kind of... ensnare you in a kind of isolating mental practice.

P11

One participant was conscious of limiting their reliance on technology when accessing mental health support to challenge avoidance behaviors linked to their mental health difficulty:

So, for me, I prefer physical appointments even though I hate the agoraphobia..., but it’s important for me to still do that otherwise I won’t go out.

P8

#### Financial Implications

Before engaging with the scheme, 7 (58%) participants cited the unaffordability of technology as a reason for being digitally excluded. While participants did not have to pay to access the scheme, P7 noted that they had decided to invest in Wi-Fi when they knew they were going to be loaning a tablet. P6 noted that if there had been any extra cost involved, it would have led them to reconsider their involvement. A total of 2 (17%) participants reported that the scheme had potentially saved them money:

Yes, I’m, I have listened to certain pieces of music, in the past I would have you know, saved up for a few weeks and then bought a couple of CDs, for example, yeah.

P11

I guess that aspect of it has saved money. So, and like sending letters and replying and tracking stuff like- using my phone or my travel money.

P8

However, accessing the scheme provided other financial advantages; for example, it enabled P8 to manage their money on the web:

I use the internet banking, so it’s been paramount and stuff so. ...As well, my bank is Co-Op, so they’re very few and far between. That’s not even one close to me, so when it comes down to banking, internet is vital.

P8

Meanwhile, P1 and P2 reported that the scheme was a stopgap, enabling them to access a device, while they saved to purchase their own:

I couldn’t afford to purchase a new, a new device.... It’s given me that time to sort of bridge that gap and so I can afford to buy myself something when the loan finishes.

P1

P2 noted that after their involvement in the scheme was over, they hoped to purchase a device themselves or obtain one via a charity or other means:

I put away like £5 away each week. Just to think, maybe I can get a tablet as well, you know...I would like to have one.

P2

#### Empowerment

For most participants, the scheme provided a sense of empowerment. More than half of the participants (7/12, 58%) reported that using a tablet and having access to the digital world was either a new experience or one that broadened their world. One participant described the amount of content on the web described as a “revelation” (P11). This led participants to feel like the scheme had enabled them to learn new skills, whether it was how to access music on the web, how to use a tablet (ie, rather than a computer or laptop), or how to access the internet more generally. As a result, a quarter of participants (3/12, 25%) described that access to a tablet, connectivity, and skills now allowed them to have more control over their health, for example, by looking up information about medications, tests, and health issues or by organizing physical health appointments at the hospital, general practitioner, or dentist:

[I was] writing my appointments down on pieces of paper and things like that, so yeah. It’s a lot to manage. Like I’ve had at least 4 appointments each week for the past three months. And that’s like other- dentist, doctors, psychiatry and hospital tests...I’ve been able to manage myself a little bit better [using the tablet].

P8

Participants also felt empowered by being able to independently manage their finances (eg, web-based banking [P8]), access information (eg, health care [P7] and government services and benefits [P11]), or practical support (eg, web-based shopping [P1] or transport apps [P11]):

[What] we have done though is look up things like pensions, changes to universal credits, whether we qualify for all these new things that are coming up. So, information might change on the government websites.

P10

Participants were unanimous in reporting that the scheme was of high value and that other trusts should look to implement similar schemes:

Well, I think, I think it’s, well it’s the best thing...I don’t think that we’ve ever really had anything like this happen to us before and it has made our lives very different and a lot more interesting for both of us. And I do think that if other trusts did it, I think it’s a wonderful idea.

P7

P6 also reflected on their concern that the scheme was only available as a result of COVID-19, with digital exclusion predating COVID-19:

I don’t know if I’d have been given [a tablet], if COVID had never happened. So, I guess that’s a factor. And it might be a factor in the future for you lot. I mean the Government love to make excuses as to why they can’t find money.... Yeah, it’s hypothetical for me to kind of, put in a negative, I mean it’s a hypothetical negative, right? I wonder what my life would have been like without it. And was COVID instrumental in me having it? That’s the question I would ask.

P6

One participant highlighted how it gave service users the opportunity to engage with novel technology and build their confidence:

To me [the loaned device] was just a better option than us going to a shop and getting one and not having the first idea even how to switch it on [ ...] Without just having a little bit of help which we got, we wouldn’t really have much idea...perhaps you’re a bit shy about getting into new technology...and I think without being pushed into it, we might never have actually, you know, ever thought we, we would possibly even attempt to use one.

P7

## Discussion

### Principal Findings

This study highlighted the experience of digitally excluded service users engaging in an innovative tablet loan scheme. The scheme aimed to mitigate the detrimental impact of digital exclusion on service users who lacked the necessary equipment, skills, or confidence to access mental health services or effectively engage with digital ways of working at a societal level.

Findings align with prior research on the 3-level digital divide, emphasizing the negative consequences of COVID-19–related restrictions on health service access and well-being of individuals with preexisting mental health difficulties [18]. Semistructured interviews revealed 10 major themes describing the facilitators and barriers to engagement with the scheme and subsequent impact on well-being. Factors limiting engagement included disability; poor mental health; financial constraints; and concerns around web-based safety and conditions of the loan device use, linked to lower skills and confidence. This aligns with prior research on the limited uptake of digital inclusion initiatives [12,40]. While some participants additionally reported a belief that older age influenced support needs, it was not within the scope and nature of this study to specifically assess whether age determined technological ability. However, the expressed views highlight the importance of tackling stereotypes and providing enhanced digital support for older adults, where required [41,42].

Factors facilitating engagement with the scheme included the relationship to referrers and the wider organization, as well as the personalized approach to providing digital support. Staff played a crucial role facilitating access to the scheme, but motivation varied among participants. Most expressed a desire to learn and understand technology, while others wanted to use the device to access basic functions. Some felt obligated to accept the help due to their relationship with their health care professionals, others explicitly chose not to engage with digital technology or the scheme, challenging the assumption of digital inclusion initiatives providing a desired solution. Moreover, most participants described lacking awareness of the scheme before being referred, which may be attributed to communication being limited to trust staff initially to manage limited capacity and resources before advertising the scheme more widely through posters and trust-wide communications. Regardless of their own level of engagement or participation in the scheme, participants unanimously emphasized the importance of the DIO. Participants also praised the overall value of the scheme and suggested that other NHS trusts would be well-serviced in implementing similar schemes. All participants described the wide-ranging impact the DIS had on their lives; facilitating the provision of mental health and other services; enabling participants to take control of their health care and finances; and providing access to social, educational, and entertainment opportunities. Participants also reported an improvement in well-being and gaining a sense of empowerment, without incurring additional costs. However, limited resources and the time-limited nature of the loan constrained the support offered by the DIS, a concern also seen in other initiatives limited in time or reach [4,12,42].

### Lessons Learned

Understanding service user motivation and needs is crucial for tailored support, ensuring it is relevant and not presumptive. Future initiatives should prioritize individual consultation before implementation of schemes, even if time-consuming, to ensure full engagement and use of the support offered [43].

A dedicated DIO is a vital resource, both in administering the scheme and safeguarding service user needs. It was necessary to ensure that there was a thorough risk assessment as well as to facilitate a person-centered approach. Given the complexity of mental health needs, the DIO in turn benefited from ad hoc clinical supervision to manage risk and ensure that service user needs were met sensitively and appropriately.

Several participants noted reluctance or concern around using the loan device fully as it was not their own. Gifting devices may allow for greater engagement or use; however, finite resources were a major limitation in the scheme. Evaluations such as this highlighting the benefits of digital inclusion projects across health care services are vital to demonstrate the value of investment [44].

Considering the specific use and accessibility of loan devices is critical. It may increase engagement if the service users’ specific needs were considered to determine which equipment to provide (eg, laptop rather than tablet) and the duration and type of support required to prevent the subsequent reexclusion.

Schemes should be tailored to those who need and want it most. service users who are digitally excluded will not have access to web-based promotion or lack in understanding the value of digital technology, thus not feeling compelled to ask for support. It may be helpful to be creative in increasing awareness through physical means (eg, posters or leaflets) and workshops and by advertising support offers across community services [42]. It is also helpful to connect with staff, for example, by attending team meetings, to highlight the value of the scheme and thus facilitate the access for service users under their care.

### Limitations and Future Considerations

Challenges in evaluating patient-centered programs include biased sampling or unreliable self-report data [18,43]. In addition, the method of contacting participants exposed digital divides, as those unable to opt in on the web had fewer opportunities to share their views, while the influence of health factors may have impacted others’ ability to participate [11]. Moreover, by using a convenience sample drawn only from the service users who had previously engaged in the scheme, there was a risk of social desirability bias, with participants providing more positive evaluations, while insights from service users who were unable to or did not want to engage with the scheme were not captured. To address these limitations, the interviewer ensured that all the participants were aware that both positive and negative evaluations were welcome and would not negatively impact their current or future access to the scheme. In addition, the qualitative nature of the study did not allow for an investigation into the causal relationship between specific factors and digital exclusion. Nevertheless, understanding the demographics, motivations, and challenges experienced by those who participated in the scheme provided valuable insights into the populations that require support and those who may need more targeted interventions [42].

Finally, one might query whether the need for digital inclusion initiatives is still required, given the lessened immediate impact of COVID-19–related disruption on services. However, while this scheme was introduced in the height of COVID-19, the NHS long-term plan had long recommended a continued expansion of digital transformation initiatives across the United Kingdom [9]. Mounting evidence compiled before, during, and after COVID-19 suggests that web-based therapeutic interventions are at least equivalent to in-person alternatives [7] and are increasingly being trialed and evaluated in specialist health care services, such as prisons [45]. Outside of the NHS, many essential public services have also moved on the web, with the UK government committing to increased spending and investment to further improve efficiency and ease of access [46].

But despite the wider financial benefits of implementing web-based services [47], many still express a desire for in-person availability of support [47]. Furthermore, nearly a quarter of working-age adults report living with a disability that impacts their day-to-day functioning [48], alongside greater prevalence of mental health conditions and lower employment rates [49]. Given the disproportionate impact on these groups [50], it will be necessary to consider the impact of digital transformation initiatives on access to health care, public services, and wider society for those who are not digitally enabled.

The debate around who should bear the responsibility of digital inclusion initiatives continues, with community organizations and libraries often serving as popular suggestions [44,51]. Others have argued for interventions on a macrolevel through policy change [24,25]. However, in this paper, we present the notion that when presented with disrupted access to health care, providing support at source, that is, by the health care provider itself, can have a profound impact on uptake and response.

### Conclusions

This evaluation aimed to demonstrate the potential of a digital inclusion initiative to enable NHS mental health service users to access the digital world. By examining engaged service users and their experiences within the scheme, valuable insights have been gained, addressing a previously existing knowledge gap. The study offers recommendations for future digital inclusion efforts while also highlighting the importance of providing equitable access to digital technology and support within health care contexts, especially given the rise in remotely delivered health care services.

## Appendix

Multimedia Appendix 1 Camden and Islington National Health Service Foundation Trust Digital Inclusion Project (baseline survey).

Multimedia Appendix 2 Semistructured interview schedule.

Multimedia Appendix 3 Participant information sheet.

Multimedia Appendix 4 Camden and Islington National Health Service Foundation Trust Digital Inclusion Scheme evaluation consent form.

## Abbreviations

C&I NHS FT: Camden and Islington National Health Service Foundation Trust
DIO: digital inclusion officer
DIS: Digital Inclusion Scheme
ICT: information and communications technology
NHS: National Health Service
