# Supplementary material for: Impact of Digital Inclusion Initiative to Facilitate Access to Mental Health Services: Service User Interview Study
Source: JMIR Ment Health. 2024 Jul 26;11:e51315. doi: 10.2196/51315 (PMC11316150; doi:10.2196/51315)
Supplement: Multimedia Appendix 1 [file mental_v11i1e51315_app1.docx]

## **Multimedia Appendix 1**

**C&I Digital Inclusion Project (DIP) - Baseline survey**

Thank you for taking the time to fill in the following survey.

By completing the questionnaire, you are giving your consent for us to use the information you provide for research purposes. The results will help us to better understand the impact of digital exclusion on your access to service and how the provision of Camden and Islington NHS Digital Inclusion support can help with this.

You are not obliged to fill in this survey, and your support or care will not be affected if you decide not to. If you do fill it in, you can change your mind later and ask us to arrange to have the information deleted from our records. The information you give will be stored on by the Camden and Islington NHS Trust.

We do not ask for any personally identifiable information and only use anonymous aggregate data when presenting the findings. This ensures that information provided is kept private and confidential. All the information you give will be kept securely and handled in accordance with the Data Protection Act 2018 and the General Data Protection Regulation (EU) 2016/679.

If you have any questions, please do not hesitate to speak to us. You also have the right to withdraw your consent at any time by speaking to a member of staff at the Camden and Islington NHS Foundation trust by phone or in person (St Pancras Hospital, 4 St Pancras Way, London, NW1 0PE).

**Section 1 - General Information**

1.Name *[Required to answer. Single line text].*

Enter your answer: ____________________________________________________________

**Section 2 - Use of digital technology and/or internet**

How have you been using the internet or digital technology available to you at present.

2.Do you consider your digital skills to be: *[Required to answer. Single choice.]*

- Good
- Average
- Poor

3.How confident are you in using digital technology: *[Required to answer. Single choice.]*

- Extremely confident
- Somewhat confident
- Neutral
- Somewhat not confident
- Extremely not confident

4.How regularly do you use the internet? *[Required to answer. Single choice.]*

- Every day
- A few times a week
- A few times a month
- Never

5.Is there anything that limits you from using the internet at the present moment? Select all that apply. *[Required to answer. Multiple choice.]*

1. I did not have a device
2. I did not have an internet connection
3. I had an internet connection but it was too slow
4. My device was not good quality
5. There was no reason for me to use my device
6. None of my friends and family are online
7. I struggle to know how to use my device
8. I do not have time
9. Other

6.What do you use the internet to do? *[Required to answer. Multiple choice.]*

- Nothing, I didn't use it
- To stay up to date (i.e. read the news or Twitter)
- For entertainment (i.e. to watch videos)
- To learn
- To earn money
- To do online shopping
- To speak to friends and family
- To speak to my C&I clinician, care coordinator or service
- To access additional mental wellbeing support, such as online information and apps
- To speak to my GP
- To access other physical and/or mental health services
- To access social services
- Other

7.Has access to digital technology and internet become more important to you during the Covid-19 pandemic? *[Required to answer. Single choice.]*

- Yes
- No

8.If yes, how has it become more important to you during the Covid-19 pandemic? *[Required to answer. Single line text].*

Enter your answer: __________________________________________________________________

**Section 3 - Digital Support**

9.Which of the following do you think you'll need support with from the C&I Digital Inclusion Project? Please select all that apply. *[Required to answer. Multiple choice.]*

- A device (tablet/laptop)
- IT support from Ability Net
- Connectivity support to set up wifi/broadband
- Support/advice from the Digital Inclusion Officer
- All of the above
- Other

10.Is there anything you think we should know that would help us provide you with the best possible digital support? *[Required to answer. Single line text].*

Enter your answer: __________________________________________________________________

Thank you.
