# Supplementary material for: Impact of Digital Inclusion Initiative to Facilitate Access to Mental Health Services: Service User Interview Study
Source: JMIR Ment Health. 2024 Jul 26;11:e51315. doi: 10.2196/51315 (PMC11316150; doi:10.2196/51315)
Supplement: Multimedia Appendix 2 [file mental_v11i1e51315_app2.docx]

## **Multimedia Appendix 2**

**Semi-Structured Interview Schedule**

**Participant Code:**  **Date:** **How recorded:** F2F / TC /VC

**Introduction**

The purpose of this interview is to explore your experience of accessing the Digital Inclusion Scheme at Camden and Islington NHS Foundation Trust.

This is a semi-structured interview; this means I have a list of questions to explore, however, the conversation is flexible so please feel free to talk openly while I may also ask follow-up questions, where appropriate.

The interview should take approx. 50 minutes. Please let me know if you would like to take a break or need to stop at any time. You can change your mind about taking part in this evaluation at any time, by letting me know or by contacting Dr Julia Gillard, Clinical Psychologist (t: 0203 317 6820, email: [Julia.Gillard@candi.nhs.uk](mailto:Julia.Gillard@candi.nhs.uk)) or the Digital Inclusion Officer (DIO) (t: 02033177107, email: [dio@candi.nhs.uk](mailto:dio@candi.nhs.uk)). You can also write to the Digital Inclusion Scheme located on the 4^th^ Floor, West Wing, St Pancras Hospital, Camden and Islington NHS Foundation Trust, London, NW1 0PE.

If you are happy to proceed, I will record this interview session so that I can transcribe and analyse your responses, which will be used for funding, reporting, and dissemination purposes.

Once your answers have been transcribed, the recording will be deleted. Your response will be kept confidential and anonymised to protect your identity. This means that your name and any personal reference you make to specific people or places will be removed so you cannot be identified.

The results of the service evaluation will be written up and will be used for evaluation reports and to inform improvements to the scheme. The report will be shared with and may be presented to staff within C&I. Anonymised data will be analysed by Anglia Ruskin University. Where possible, we will also aim to publish the results in academic journals to further disseminate the findings of this study. However, no identifiable information will be used and there will be no publicly available record that can link your data to the findings presented.

We do not anticipate that there are any risks associated with taking part in this interview however you may stop the session at any time or skip questions you do not wish to answer. At the end there will be the opportunity to ask any questions or voice any concerns.

Do you have any questions before we start?

[Interviewer checklist

- check consent form has been signed and any outstanding questions have been addressed
- check audio-recording and/or transcription is on
- prompts are provided in italics]

**OUR SERVICES**

Before we get started, could you tell me which services you have accessed

- Digital Needs Assessment with DIO
- Tablet Loan
- Internet connectivity router (Jangala)
- Mobile Data (Vodafone)
- Digital Skills Support referral (AbilityNet)

**General Background:**

1. In your own words, what do you think is meant by the term digital inclusion/exclusion?
   There is no right or wrong answer.
2. What led you to being referred to the C&I Digital Inclusion Scheme?
   - *Were you aware that you were being referred?*
   - *Experience beforehand - skill/motivation/confidence/access?*
   - *Impact on exclusion on life?*
3. What were your motivations/hopes/expectations when engaging with the scheme?

**Primary Outcomes**

1. Can you tell me about your experience of accessing the C&I Digital Inclusion Scheme?
   - *Likes/dislikes?*
   - *Quality of support? (e.g., suitable internet speed)*
   - *Skills developed?*
2. What helped or hindered (barriers) you in engaging with the support offered by the scheme?
   - *What made it difficult for you? How did you overcome this?*
   - *What made it difficult for me when working with this person?*
   - *Is there anything more we could have done to support you with this?*
   - *Additional costs incurred? (e.g., WiFi, broadband, online subscriptions, scams)*
3. How has engaging with the scheme enabled you to access (mental health) services at C&I?

**Partnerships: GetBox/Vodafone/LTE (specify in conversation); AbilityNet**

1. Can you tell me about your experience of using the Jangala GetBox (internet connectivity device/router)?
2. What benefits, if any, has free data access (with Vodafone SIM) to the internet brought you or your household.
3. Can you tell me about your experience of getting digital support via AbilityNet?

**Secondary Outcomes**

In what way has the scheme had an impact on your well-being (long or short term)?

*Can you give me an example?*

*Mental/physical/general wellbeing?*

1. What impact has the scheme had on your ability to engage with the digital world more generally?
   - *Skills, motivation or confidence when engaging with the digital world?*
   - *Is there anything you still find challenging about engaging with the digital world?*
2. What other services/resources do you now access online, if applicable?

- *Health, community, or public services?*
- *Access to mental health services, individual or group-based interventions?*
- *Going online to find information about health-related issues or medical care?*
- *To make an appointment with a doctor or health practitioner or order repeat prescriptions?*
- *Use online services to apply for housing/benefits?*

1. Has accessing the scheme changed the way you spend your time or connect with others?

- *Keeping connected social relationships? (e.g., if I want to, there are people I can talk to online if I’m feeling lonely; use of video calling, online groups etc)*
- *Leisure time? (e.g., go online to find information about leisure activities, including cinema, arts, live music and other events, such as holidays)*

1. What, if any, financial implications have there been as a result of accessing the scheme?
   - *Managing/saving money? (e.g., buying things online; accessing online banking; bill payments etc)*
   - *Financial savings? (e.g., by saving on transport costs/other personal expenditure, out of pocket costs like work hours missed etc)*
   - *Additional costs? (e.g., WiFi/data etc)*
2. Has the scheme provided any other opportunities?

- *Education? (e.g., to do online distance learning for an academic degree or job training)*
- *Employment? (e.g., to find/apply for a job through the internet, able to secure job through having gained IT skills)*
- *Access to goods and services? (e.g., purchasing/selling etc)*
- *Any additional digital support? (e.g., Recovery College)*

1. What, if any, value do you think is there in NHS trusts implementing Digital Inclusion initiatives like ours?
2. Is there anything else you would like to add?

**About you**

We want to make sure our services are accessible and inclusive. Telling us a little about yourself will help us make sure they are working for everyone. If we find that some people are not using our services or it is not helping them as much as we had hoped, we can make changes to improve this.

Do you mind if I ask you a few demographic questions?

1. **How old are you?**

- 18-24
- 25-34
- 35-44
- 45-54
- 55-64
- 65-74
- 75-84
- 85+

1. **What best describes your gender?**

- *Male*
- *Female*
- *Non-binary*
- *Transgender Female*
- *Transgender Male*
- *Gender-Fluid / Gender-Queer*
- *Intersex*
- *Prefer not to say*
- *Other*

1. **What best describes your ethnicity?**

- White British
- White Irish
- White - any other White background
- Asian or Asian British Pakistani
- Asian or Asian British Indian
- Asian or Asian British Bangladeshi
- Any other Asian background
- Black or Black British - Somali
- Black or Black British – Caribbean
- Black or Black British - African
- Black or Black British - Any other Black background
- Mixed White and Black African
- Mixed White and Black Caribbean
- Mixed White and Asian
- Mixed - any other Mixed background
- Other Ethnic Groups – Chinese
- Other Ethnic Groups – Turkish
- Other Ethnic Groups – Any other Ethnic Group
- Not Stated
- Not Known
- Other _______________________

1. **What is your current employment status?**

- Full-time employed
- Part-time employed
- Self employed
- Unemployed
- Unable to work
- Student
- Retired
- Other: __________________________
- Prefer not to say

1. **Which of these is the highest educational institute you have attended?**

- Primary school
- Secondary school
- Sixth form college or further education (A-levels, BTEC, etc.)
- Higher education or University (diploma, Bachelor etc.)
- Post-graduate education
- Prefer not to say

1. **Do you consider yourself to have disability/long term (mental) health condition?**

- Yes
- No
- Prefer not to say

1. **Which of the following best describes your relationship status?**

- Married/civil partnership
- Living with someone, but not married or in civil partnership
- Single
- Divorced or separated
- Widowed
- Prefer not to say

1. **Which of the following best describes your household income last year?**

- Below £10, 000
- £10, 001 to £20, 000
- £20, 001 to £30, 000
- £30, 001 to £40, 000
- £40, 001 to £50, 000
- Above £50, 001
- Prefer not to say

Thank you very much for taking part in this interview. Do you have any questions before we finish?

If you need to speak with us about anything related to this interview or the Digital Inclusion Scheme, then please don’t hesitate to get in touch with us by telephone on 02033177107 or by email on [dio@candi.nhs.uk](mailto:dio@candi.nhs.uk).

If you feel you need support around your mental health, then please contact your GP or health professional at Camden and Islington NHS. If you do not feel that you can wait to see your GP or health professional, then please contact the C&I Crisis Team on Freephone 0800 917 3333. This number is available 24 hours a day, 7 days a week.
