# Supplementary material for: Impact of Digital Inclusion Initiative to Facilitate Access to Mental Health Services: Service User Interview Study
Source: JMIR Ment Health. 2024 Jul 26;11:e51315. doi: 10.2196/51315 (PMC11316150; doi:10.2196/51315)
Supplement: Multimedia Appendix 3 [file mental_v11i1e51315_app3.docx]

## **Multimedia Appendix 3**

**Camden & Islington NHS Digital Inclusion Scheme Feedback Session**

**Information Sheet**

***What is the purpose of the evaluation?***

The Camden & Islington (C&I) Digital Inclusion Scheme is carrying out a service evaluation in the form of semi-structured interviews to better understand the experiences of service users who have accessed the Digital Inclusion Scheme. In hearing about your experience of engaging with the Digital Inclusion scheme, we will be in a better position to understand how we can improve our services and ensure more service users can benefit from support. The final report and published findings can also be used to inform best practice around patient-centred care and support other services in developing and running similar Digital Inclusion initiatives.

***Why have I been invited?***

You have been invited as you have previously accessed or are currently accessing the Digital Inclusion Scheme at C&I NHS Foundation Trust. We would like to interview you to hear directly about your experience of the scheme.

***Do I have to take part?***

Participation in the research is entirely voluntary. You may decide that you do not want to be interviewed. This will have no impact on your involvement or access to support from the Digital Inclusion Scheme.

***What will happen if I take part?***

If you chose to participate, you will be contacted by the Digital Inclusion Scheme team to arrange a time to be interviewed. The interview can be over the phone, by video-call, or in person. With your permission, these interviews will be audio recorded for the purpose of transcription. This process allows your feedback on the service to be analysed more easily. If you agree for your interview to be recorded but feel at any stage that you would like to stop the recording, you are free to ask me so at any time. After your interview, your personal data will be anonymised. The anonymised feedback will be included in the evaluation report and inform service improvements. All recorded material will be destroyed once the results have been written up.

***Will my details remain confidential?***

Your personal data and responses will be kept confidential in line with the UK General Data Protection Regulation (UK GDPR) and the Data Protection Act (2018). Only authorized members of the Digital Inclusion Scheme at Camden and Islington NHS Foundation Trust will have access to your responses. Once transcribed, the data collected will be made anonymous to protect your identity. This means your name and any personal reference you make to specific people or places will be removed so you cannot be identified. There will be no record that links the data collected from you to personal detail, which you could be identified from. For further information on the C&I NHS Foundation Trust GDPR policy, see here: <https://www.candi.nhs.uk/about-us/how-we-handle-your-information>. This general privacy notice explains the use of your personal data for evaluation purposes.

***What will happen if I no longer want my responses to be used for the service evaluation?***

You are free to withdraw from the evaluation at any point before, during, or immediately after the interview. Once we have anonymised the responses, you will unfortunately no longer be able to withdraw your data as we will no longer be able to link responses to individual participants.

Your participation or non-participation in the feedback session will not impact on your involvement with or access to support provided by the Digital Inclusion Scheme.

To withdraw your consent, you can speak directly to any member of the Digital Inclusion Scheme, call us on 02033177107, or email us [dio@candi.nhs.uk](mailto:dio@candi.nhs.uk). You can also write to the Digital Inclusion Scheme at 4th Floor, West Wing, St Pancras Hospital, Camden and Islington NHS Foundation Trust, London, NW1 0PE.

***What will happen to the results of the service evaluation?***

The results of the evaluation will be written up and will be used to prepare a report and to inform improvements to the scheme. To do this, the anonymised responses will be analysed by researchers at Anglia Ruskin University. The final report will be shared with Camden and Islington NHS prior to submission and may be presented to staff and service users within C&I. Where possible, we will also aim to publish the results in academic journals to further disseminate the findings of this study. However, no identifiable information will be used and there will be no publicly available record that can link your data to the findings presented.

***Who has reviewed the service evaluation?***

This service evaluation has been registered with the Camden and Islington Governance and Quality Assurance team.

**Who will be carrying out the evaluation?**

The principal investigators are Dr Julia Gillard (Clinical Psychologist, C&I NHS Foundation Trust and Senior Lecturer in Clinical Psychology, Anglia Ruskin University), Ms Amy Oliver (Honorary Digital Inclusion Officer, C&I NHS Foundation Trust and Research Assistant, Anglia Ruskin University) and Ms Ella Chandler (Digital Inclusion Officer, C&I NHS Foundation Trust).

***What are the possible benefits of taking part?***

The information you provide will form a valuable contribution to the Digital Inclusion Scheme. Your responses will contribute to a greater understanding of the service and its limitations and will inform future improvements. It may also support other organisations in developing and implementing their own Digital Inclusion initiatives to support individuals at risk of digital exclusion.

***What are the possible disadvantages and risks of taking part?***

Some people can find it upsetting to answer questions about their personal experiences. We will support you if you become upset and will let your referrer into the Digital Inclusion Scheme know so that they can also speak with you, should you have found discussing your experiences difficult.

If you feel you need further support, then please contact your GP or health professional. If you do not feel that you can wait to see your GP or health professional, then please contact the C&I Crisis Team on Freephone 0800 917 3333. This number is available 24 hours a day, 7 days a week.

***What if there is a problem?***

If you wish to contact the Digital Inclusion Scheme team to discuss any of the above or if you any concerns about the service evaluation, then please do so by getting in touch with Dr Julia Gillard, Clinical Psychologist (telephone: 0203 317 6820, email: [Julia.Gillard@candi.nhs.uk](mailto:Julia.Gillard@candi.nhs.uk)) or the Digital Inclusion Officer (DIO) (telephone: 02033177107, email: [dio@candi.nhs.uk](mailto:dio@candi.nhs.uk)). You can also write to the Digital Inclusion Scheme at 4th Floor, West Wing, St Pancras Hospital, Camden and Islington NHS Foundation Trust, London, NW1 0PE.

**Thank you for taking the time to read this information sheet.**
