# Supplementary material for: Impact of Digital Inclusion Initiative to Facilitate Access to Mental Health Services: Service User Interview Study
Source: JMIR Ment Health. 2024 Jul 26;11:e51315. doi: 10.2196/51315 (PMC11316150; doi:10.2196/51315)
Supplement: Multimedia Appendix 4 [file mental_v11i1e51315_app4.docx]

## **Multimedia Appendix 4**

**C&I Digital Inclusion Scheme Evaluation**

**CONSENT FORM**

| Have you been given enough information explaining the purpose of this feedback session? | Yes/No |
| --- | --- |
| Have you had enough opportunity to ask questions and discuss this feedback session? | Yes/No |
| Have you received satisfactory answers to all questions you asked? | Yes/No |
| Have you received enough information about the evaluation for you to make a decision about your participation? | Yes/No |
| Do you understand that you are free to withdraw from the evaluation and free to withdraw your data at any time prior to anonymisation and without having to give a reason for withdrawing? | Yes/No |
| Do you consent to the interviews to be audio recorded for the purpose of transcription? | Yes/No |

**I hereby fully and freely consent to my participation in this feedback session**

I understand the nature and purpose of the procedures involved in this session. These have been communicated to me on the information sheet accompanying this form.

I understand and acknowledge that the aim of the evaluation is to provide feedback of my experience of the C&I Digital Inclusion Scheme to evaluate the impact of the project so that the service to make improvements and disseminate the findings so other Trusts may benefit from the lessons learnt.

I understand that the data will be kept **confidential**, and that on completion of the session my data will be anonymised by removing all links between my name or other identifying information and my interview responses. This will be done before the data is written into a report and before any presentation or publication of my data. All recorded material obtained for the purpose of transcription will also be destroyed at this point.

Participant’s signature: ___________________________ Date: _______________

Name in BLOCK letters: ___________________________

If you have any questions about the service evaluation, please contact Dr Julia Gillard, Clinical Psychologist (t: 0203 317 6820, email: [Julia.Gillard@candi.nhs.uk)](mailto:Julia.Gillard@candi.nhs.uk) or the Digital Inclusion Officer (DIO) (t: 02033177107, email: [dio@candi.nhs.uk](mailto:dio@candi.nhs.uk)). You can also write to the Digital Inclusion Scheme at 4th Floor, West Wing, St Pancras Hospital, Camden and Islington NHS Foundation Trust, London, NW1 0PE
